# A rare case of renal infarction due to heroin and amphetamine abuse: case report

**DOI:** 10.1186/s12882-021-02642-1

**Published:** 2022-01-12

**Authors:** Suhail Khokhar, Daniela Garcia, Rajesh Thirumaran

**Affiliations:** 1 PGY-2 Internal Medicine Resident at Indiana University Southwest, Evansville, USA; 2 PGY-3 Radiology resident at Mercy Catholic Medical Center, Philadelphia, USA; 3 Hematologist/Oncologist at Mercy Catholic Medical Center, Philadelphia, USA

## Abstract

**Background:**

Renal infarctions as a result of recreational drug use are rare and are commonly associated with cocaine use. Although amphetamines have a similar mechanism of action as cocaine, there are few reports linking them to ischemic events, and only one to renal infarction. Similarly, few reports link heroin use with infarcts, but never in the kidney. Although uncommon, several mechanisms have been implicated in heroin and amphetamine-induced infarction, including vasculopathy, vasculitis and the activation of the coagulation cascade.

**Case Presentation:**

47-year-old female with a past medical history of non-intravenous heroin and amphetamine abuse, chronic obstructive pulmonary disease, hypertension, hyperlipidemia presented with right lower extremity swelling and rash, which was diagnosed as cellulitis and treated appropriately. Incidentally, the patient was found to have an acute kidney injury and further workup identified multiple renal infarcts in the right kidney. The patient had no past medical history of clotting disorders. Blood culture and urine cultures were sterile; autoimmune and hypercoagulable workup were negative. Urinalysis was unremarkable. Urine toxicology was only positive for opiates and amphetamines, which were thought to be the most likely cause of the renal infarct. Patient was lost to outpatient follow up due to noncompliance, but returned to the hospital for re-emergence of her cellulitis, during which no new infarcts were discovered, and the previous renal infarct had scarred over.

**Conclusion:**

There are very few reports of heroin and amphetamine-induced infarctions. This case report describes a rare but important complication of heroin/amphetamine abuse that could be easily overlooked.

## Background

Renal infarction occurs when blood flow to a region of the kidney is obstructed and the resultant hypoxia causes tissue damage. Atrial fibrillation is the most common risk factor for renal infarction [2]. Symptoms of renal infarction are often flank pain, nausea and vomiting. In addition to radiologic findings, an increase in lactate dehydrogenase, C-reactive protein and impairment of renal function is often noted in patients with renal infarcts [6]. There is only one report of amphetamine-induced renal infarction and none linking heroin to this complication [9]. It is estimated that over one million people abuse heroin or amphetamine in the US, making complications secondary to these drugs an important healthcare issue [5]. Renal infarction has currently limited treatment options and timely detection is of utmost importance.

## Case presentation

**Fig. 1:**
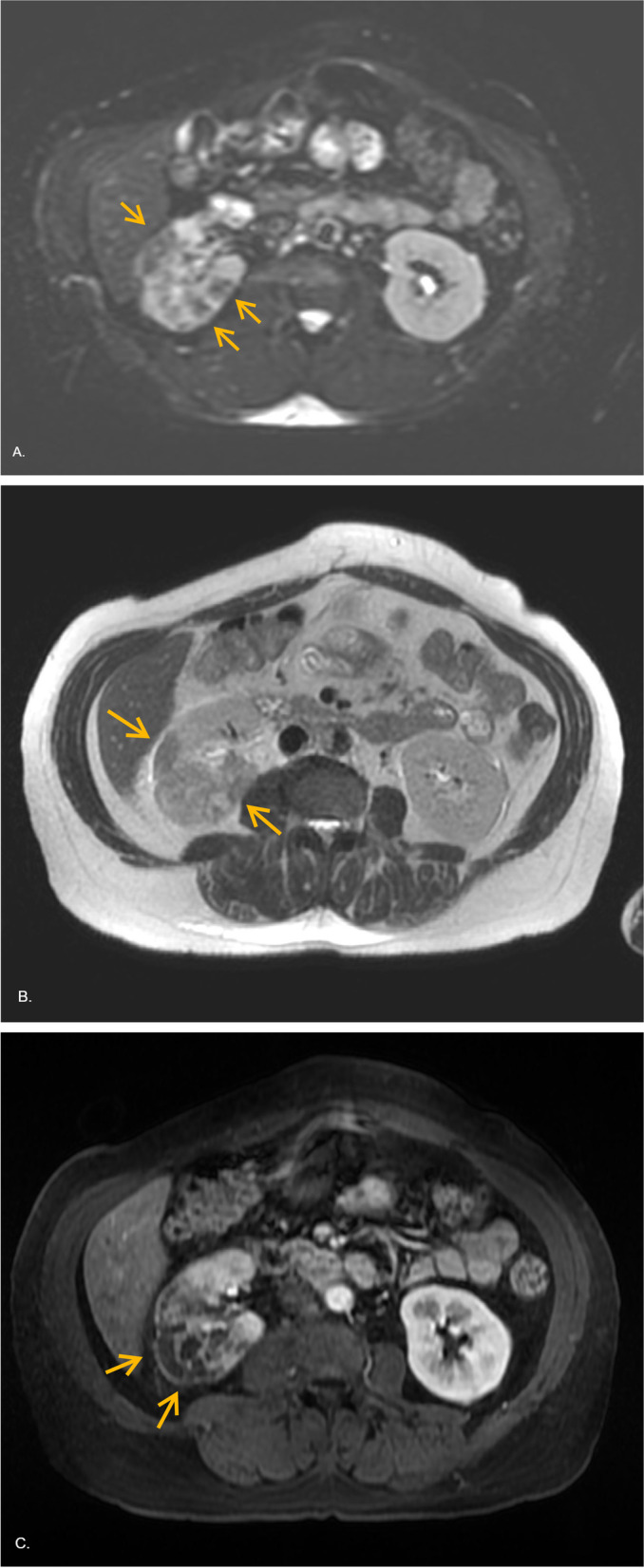
**A** DWI demonstrated no diffusion restriction (arrows). **B** T2 weighted image showed hypointense signal compared with normal parenchyma (arrows). **C** Post contrast T1 weighted image showed wedged shaped infarcted areas with low signal intensity with obliteration of corticomedullary differentiation of right kidney (arrows)

**Fig. 2:**
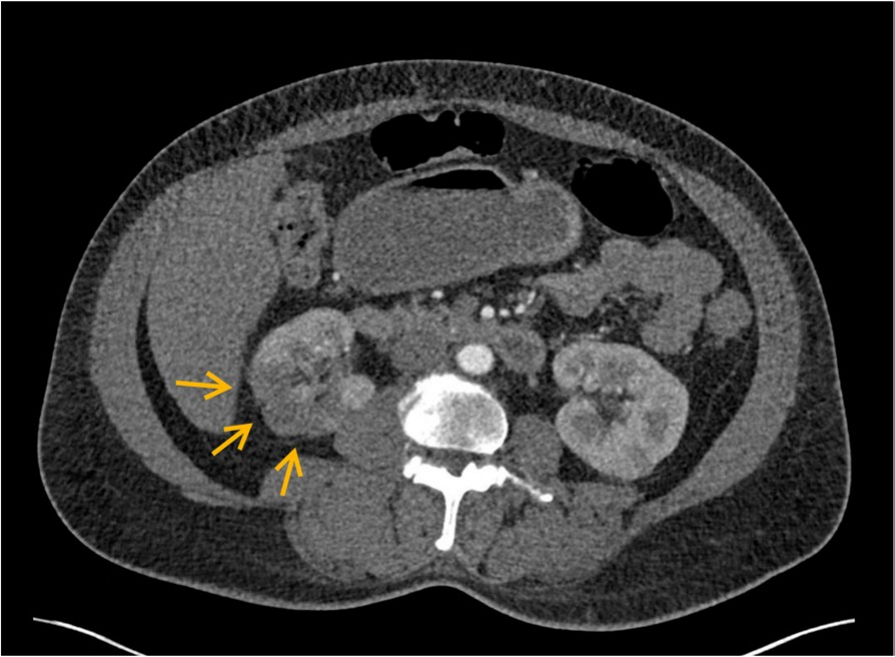
CT performed about 1 month after revealing hypodense lesions and cortical thinning of the mid and upper pole of right kidney consistent with scarring due to previous ischemic insult (arrows)

A 47-year-old woman with past medical history of non-intravenous heroin and amphetamine abuse, tobacco dependence, chronic obstructive pulmonary disease, hypertension, and hyperlipidemia presented to the emergency department with complaints of right lower extremity swelling and rash 1 week prior to arrival. Pain in the leg was reported to be recurrent. She had leukocytosis of 23.9 k/μL (79.7% neutrophils), creatinine of 2.4 mg/dL (with a baseline creatinine of 1 mg/dL), C-reactive protein elevation to 10.8 mg/L. Erythrocyte sedimentation rate was 20. She also had mild elevation of lactic acid to 2.4 and hypotension, both of which resolved after receiving a 1 l bolus of normal saline. Ultrasound of bilateral lower extremities was performed, and no deep venous thrombosis was identified, so patient was admitted for IV antibiotic treatment of right leg cellulitis. Physical exam was only significant for erythema of the right leg up to the mid shin. Blood cultures and urine cultures collected prior to starting antibiotics showed no growth. Renal ultrasound obtained to evaluate the acute kidney injury found a hypoechoic region in the right kidney. MRI of the abdomen identified multiple infarcts with the largest measuring 5 cm in the upper pole and 4 cm in the mid-pole of right kidney (Figs. 1 and 2. MRI of the abdomen was negative for malignancy and just showed benign adrenal adenoma, urine metanephrines were 60 μg/day (ref 58–203 μg/day) and catecholamines were 8 μg/day (ref 15–100 μg/day). These showed a preserved capsular enhancement. Right renal artery and vein were patent. No surrounding edema or diffusion restriction was observed to suggest an abscess. Patient did not complain of any abdominal pain or nausea. Patient denied any history of spontaneous abortions, but noted a family history of blood clots, which prompted a hypercoagulable work up. PT/INR and APTT were within normal limits. Her AST was 20 U/L (Ref 10–40), ALT was 30 U/L (Ref 7–52), total bilirubin was 0.4 mg/dl (Ref 0.2–1.2), and direct bilirubin was 0.1 mg/dl (Ref 0–0.2). Factor V Leiden mutation was not detected. The activity of protein C and S were 114 and 102, respectively, within their normal ranges. Antithrombin III activity was 85, also within normal range. Antiphospholipid antibodies and homocysteine levels were within normal limit. The chance of vasculitis in a patient with an ESR of 20 is very small, but nonetheless, workup for collagen vascular diseases was performed. The patient’s ANA screen was negative and she was also negative for double stranded DNA antibodies. ANCA serology was negative. Rheumatoid factor was negative too. Hepatitis A IgM antibody, B antigen and core IgM antibody, and C antibody and HIV p24 and antibody were negative. 2D echo was performed to rule out cardioembolic sources, and the test was without any abnormalities. EKG showed normal sinus rhythm, no atrial fibrillation, and she denied history of palpitations. She was placed on continuous telemetry and there were no episodes of atrial fibrillation on telemetry. She was diagnosed with amphetamine/heroin induced renal infarct based on exclusion of other possibilities.

The patient was asymptomatic with respect to the renal infarct. Mechanical thrombectomy was not an option considering that no large clots were discovered on imaging. Systemic thrombolysis was also not done because she was outside a likely temporal window of benefit. She was advised regarding drug cessation and was discharged with antibiotics for her cellulitis. She was advised to follow up in the outpatient hematology clinic. While she did not come to the appointment, she did return to the hospital with recurrent pain in her leg a month later. At that time, abdomen CT repeat did not discover any new infarcts, and the previous renal infarct was scarred over.

## Discussion and conclusion

Amphetamines are sympathomimetics, like cocaine, and function by increasing synaptic concentrations of monoamines [10]. They can increase heart rate as well as blood pressure via activation of the sympathetic nervous system, which is believed to be the mechanism behind hemorrhagic brain strokes [23]. Users of amphetamines also risk vascular complications such as renal artery aneurysms, renal cortical necrosis, necrotizing renal vasculopathy, or vasculitis [3, 7, 13, 19, 24]. Reports of ischemic events such as myocardial infarction are rare but are present in the literature [16, 17]. Renal infarctions due to ecstasy use, a type of amphetamine, have been reported only once [9]. Ischemic events secondary to amphetamine use is believed to be primarily driven through induction of thrombosis [20]. Amphetamines have been shown to induce tissue factor expression on the surface of endothelial cells. Tissue factor activates the coagulation pathway by binding factor VII, leading to a coagulation cascade and clot formation [12]. Additionally, amphetamines have been shown to accelerate atherosclerosis, which could further aggravate the prothrombotic state within the vessel [14]. Moreover, there is some evidence to suggest that amphetamine use can lead to vasospasm or vasculitis, which impair blood supply to the organ and creating an environment favorable for an acute ischemic event [8].

Interestingly, heroin as a drug of abuse works via a completely different mechanism than amphetamine. Heroin is an agonist of the mu, kappa, and delta opioid receptors, which upon activation can lead to a diverse set of effects such as analgesia, decreased gastrointestinal motility, and respiratory depression [15]. Despite its different mechanism of action, heroin use has also been tied to the induction of ischemic events. The biggest contributing factor to ischemia in heroin users stems from endocarditis seeding septic emboli; however, this is a complication found only in intravenously abused drugs – not the case in our patient [11]. Heroin use has also been shown to increase vascular stiffness, and there are even reports of heroin-induced cerebral and myocardial infarctions [1, 21, 22]. Dependence on opiates, a drug family which heroin is part of, has also been shown to increase fibrinogen levels, which could predispose one to a higher likelihood of an embolic event [18]. Finally, heroin has been reported to induce hyper-eosinophilic syndrome, which can also lead to thrombosis and has been reported to result in cerebral infarction [4]. In the case of our patient, her eosinophils were 200/μL (normal limit up to 700/μL), ruling out the possibility of the hyper-eosinophilic syndrome.

Our patient did not have clinical symptoms commonly associated with renal infarctions, which made a prompt diagnosis difficult and possibly limited the treatment options available for the patient. Her lack of abdominal pain could be a result of the analgesia due to heroin use. It is also possible that abdominal pain might have been overlooked in favor of the chief complaint – right leg pain.

The diagnosis of amphetamine and heroin induced renal infarct is based on the rule out of other less likely possibilities. The patient denied use of any injectable drugs and did not have signs of systemic infection leading to septic emboli. Vasculitis was very unlikely based on the very low erythrocyte sedimentation rate. Eosinophils were within normal limits, ruling out hyper-eosinophilic syndrome leading to thrombosis. Hypercoagulable workup was negative in the patient as well. It is possible that she might have an extremely rare variant that is not detected by the standard hypercoagulable workup. However, this appears to be less likely than the renal infarction being secondary to the abuse of amphetamine and heroin. Both heroin and amphetamine contribute to hypercoagulability and vasculopathy and they likely promote a sequalae leading to renal infarction. It is even possible that some of the mechanisms elicited by these drugs might even work synergistically and the combination of amphetamine and heroin is even more likely to induce ischemic events such as renal infarctions. Consideration regarding including this diagnosis on a differential in patients using both of these drugs should be made, especially since the number of amphetamine and heroin users only appears to be increasing [5].

## Data Availability

The datasets generated and/or analyzed during the current study are not publicly available due HIPAA but are available from the corresponding authors on reasonable request.
